# Retrodural space: a cadaveric evaluation

**DOI:** 10.1002/anr3.12323

**Published:** 2024-10-17

**Authors:** H. Elsharkawy, K. Lebak, A. Crofton, S. E. Pope, P. A. Traxler, S. A. Baraka, L. E. Tollinche

**Affiliations:** ^1^ Department of Anaesthesiology, Pain and Healing Center MetroHealth Medical Center Cleveland OH USA; ^2^ Department of Anatomy Case Western Reserve University Cleveland OH USA; ^3^ Department of Anatomy Case Western Reserve University Cleveland OH USA; ^4^ Department of Anatomy Case Western Reserve University Cleveland OH USA; ^5^ Department of Anaesthesiology, Pain and Healing Center MetroHealth Medical Center Cleveland OH USA; ^6^ Ohio Northern University Ada OH USA; ^7^ Department of Anatomy Case Western Reserve University Cleveland OH USA

**Keywords:** cadaver, fascial plane block, retrodural space

The retrodural space is an interfascial tissue plane located between the ligamentum flavum and the interspinous ligament [1, 2, 3]. The ligamentum flavum forms a barrier between the retrodural and epidural spaces; however, theoretically normal gaps can allow the spread of medications into the epidural space [4]. Therefore, this space can be a potential location for injecting local anaesthetics, leading to their spread into the dorsal rami, neural foramen and epidural space.

**Figure 1:**
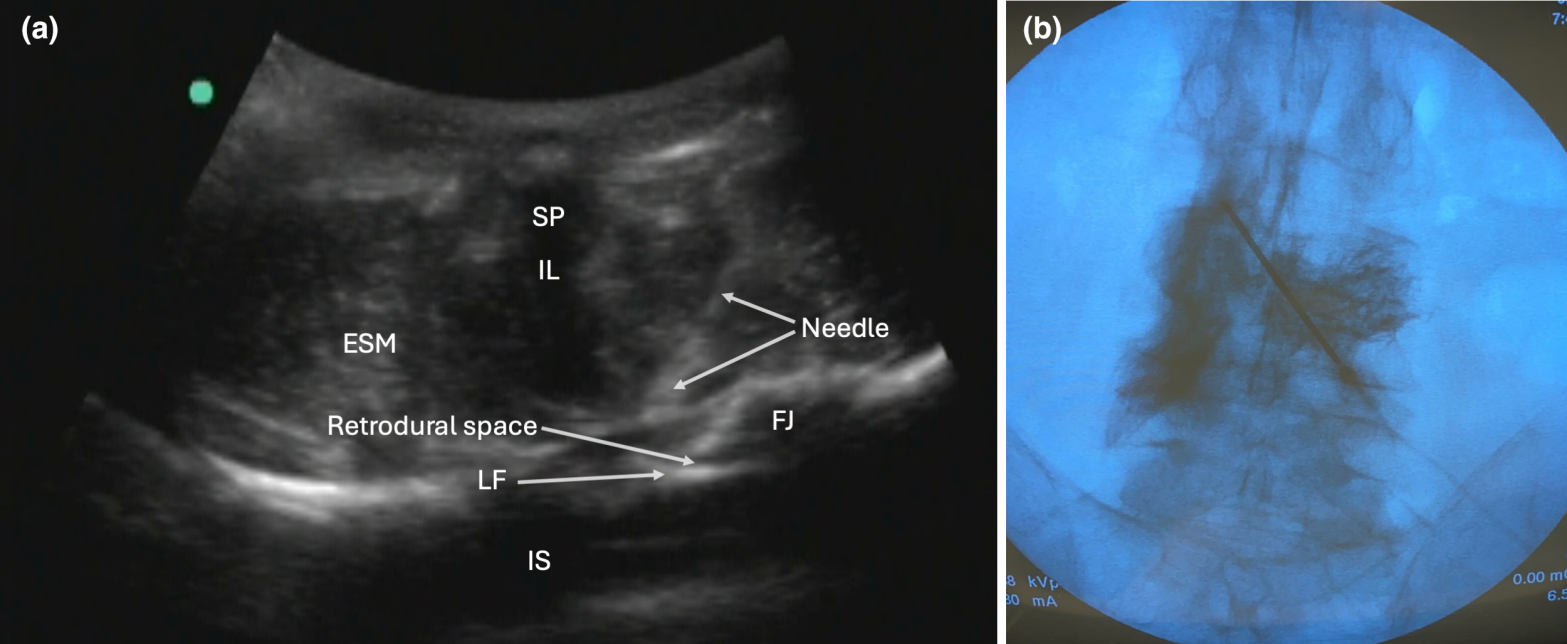
(a) Ultrasound of the needle insertion and retrodural space. (b) Lumbar X‐ray anterior–posterior view with dye contrast spread in the retrodural space. ESM, erector spinae muscle; FJ, facet joint; IL, interspinous ligament; IS, intrathecal space; LF, ligamentum flavum; SP, spinus process.

We investigated this technique in one unembalmed cadaver to determine the distribution of local anaesthetic and dye after injection into the lumbar retrodural space. Anterior–posterior fluoroscopic and ultrasound imaging (a curved array transducer in the transverse window between the L3 and L4 vertebrae) were used to guide the injection. An 18‐gauge Tuohy needle was advanced in‐plane from lateral to medial (left paramedian approach) (Fig. 1a). Once the needle tip was identified superficial to the ligamentum flavum with ultrasound and increased tactile resistance was noted, 6 ml of lidocaine 1% mixed with 0.5 ml methylene blue and 3.5 ml of iodinated contrast agent was injected (Fig. 1b).

**Figure 2:**
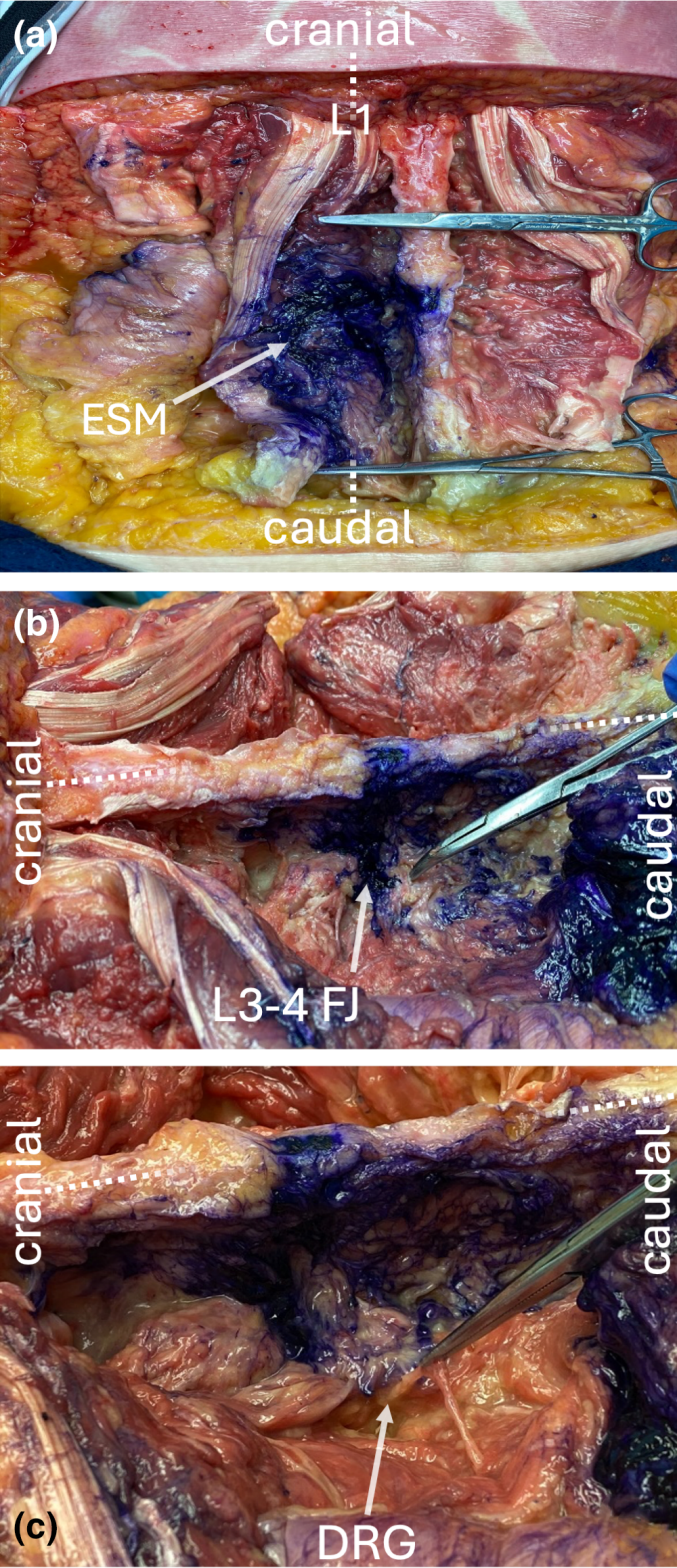
Dissection demonstrating dye spread in the cadaveric specimen. (a) Contrast deep to the erector spinae muscles from L1 to L5 levels. (b) Dissection with positive stain in the L3–4 facet joint. (c) Dissection after laminectomy and exposure of the left L3 dorsal root ganglion with no stain in the dorsal root ganglion. Dotted line represents midline. DRG, dorsal root ganglion; ESM, erector spinae muscle; FJ, facet joint.

We observed staining in the tissue plane deep (anterior) to the erector spinae muscles from T12 to L5 (Fig. 2a). The intact facet joints showed dye spread around the capsule (Fig. 2b). The lumbar dorsal root ganglion, the dura and the spinal nerves showed no staining (Fig. 2c).

This is the first cadaveric study of intentional injection into the retrodural space. This technique may prove useful for posterior truncal wall coverage as it allows the dorsal rami to be blocked.

The authors state that every effort was made to follow all local and international ethical guidelines and laws pertaining to the use of human cadaveric donors in anatomical research.
